# Neuralgia Following Fracture Successfully Treated by Operation*Read before the Fifth District Branch of the New York State Medical Association, May 26, 1891

**Published:** 1891-09

**Authors:** Reginald H. Sayre


### NEURALGIA FOLLOWING FRACTURE SUCCESSFULLY TREATED BY OPERATION.*

BY REGINALD H. SAYRE, M. D,

In the autumn of 1887 Dr. C. S. Allen brought a young man to consult me on account of an obstinate neuralgia of the right thigh. The patient was nineteen years of age, and, with the exception of an attack of small-pox when ten months old, had had good health. When five years old he fell, fracturing the right femur in its upper third. He recovered from this accident with good straight union and but little shortening. At the age of nine years he fell and refractured the right femur at a point a little higher than the former injury, and also dislocated the hip joint. The dislocation was reduced and the fracture united with a little shortening and no deformity. When thirteen years old he fractured the right femur for the third time, and this time the bones united at an angle, causing a very marked curving of the thigh outward and forward, and giving rise to between two and three inches of shortening. Since the time of the last accident he had had constant pain at the site of the injury, which became very much increased on motion, as in walking. The pain ran down the outer side of the thigh as far as the knee, and was most intense at the point of greatest deformity, which was just in the line of the external cutaneous nerve. The pain also was much increased by my manipulation of the thigh in my endeavors to locate the cause of the trouble. Of late the pain had increased so much that the patient had begun the use of morphine to secure relief.

On manipnlation of the thigh I could feel what seemed to be a ridge of bone over which a cord-like process could be slipped like a violin string, and it seemed probable that some fibers of the external cutaneous nerve or some of its branches were either pressed on by the callus or caught between the ends of the bones. Thinking, however, that the jar caused by walking with a short leg might have something to do with the pain, I advised the patient to try the effect of a high shoe on the short leg and to

*Read before the Fifth District Branch of the New York State Medical Association, May 26, 1891

blister the site of the pain thoroughly, and later on use strong galvanic currents down the course of the painful nerve, promising that I would operate on him if these means failed to afford relief.

In April, 1888, he returned again, saying that his pain had grown steadily worse in spite of treatment, and that he had been using morphine steadily. He was very much run down, his nervous system thoroughly demoralized, and I refused to operate on him until he had cut off his opium and rallied his shattered nerves.

On June 10, 1888, he was sufficiently improved to enable me to operate, which I did, assisted by my father, my brother, Dr. Allen and Dr. Carlisle. All examined the case, and all thought there was a large exostosis pressing on the nerve. Under antiseptic precautions I cut down on the most prominent part of the curved femur, and found to my surprise that part of the vastus externus muscle was so twisted on itself as to run at right angles to the long axis of the femur. I cut down to the bone and was surprised to find no exostosis with the exception of a most minute point which could hardly be considered abnormal, but which I nevertheless removed. I then passed my finger completely around the femur, stripping up the muscles for an extent of two or more inches, hoping to find some sharp projection to account for the pain; but failing to find anything, sewed up the wound with a rubber drainage-tube at the lower angle of the wound, having many misgivings as to the benefit I had done to the patient. On the second day I removed the drainage-tube, and on the fourteenth removed all the dressings, the wound being healed, and the patient having had no rise of temperature.

At first he complained of some soreness in the leg, but the old pain, on movement of any kind, disappeared from the time of the operation. When the soreness from the operation had ceased, in about a fortnight, the patient said that he felt well, and on being allowed to walk about, had none of his old stiffness and neuralgia. He left for home a month after the operation, and I have not seen him since, but received a letter from him dated January 29, 1891, in response to an inquiry after his health, in which he says: "The result so far is perfectly satisfactory. I have had no pain, and now I am able to walk and go all over without any trouble."

This case seemed to me to present points of sufficient interest to be recorded: 1. The apparent relation of small-pox in infantile life to the subsequent fragility of the right femur, which was broken in very nearly the same place on three separate occasions, each four years from the other, and caused by slight violence. 2. The persistent neuralgia following the last fracture, and persisting for six years up to the time of operation in spite of various measures that had been tried, both by myself and by others, for its relief, and which finally became so intense as to give rise to the opium habit. 3. The absence of an exostosis at the time of operation sufficient to have given rise to the symptoms, and the fact that relief was afforded by the operation. 4. The simulation of an exostosis by what I presume was a tense fiber of the fascia lata which had become so bound down as to press on the muscles of the thigh, and, by girdling them, caused the pain.

I do not clearly understand how to account for the relief of pain. I do not think the point of bone I removed was large enough to have made the trouble, and I did not think when I passed my finger around the femur that I tore loose any nerve fibers from the cicatrix. I suppose the explanation is to be found in the relief of tension given by splitting up the fascia lata, which certainly bound the muscles very tightly, and I think the length of time that has elapsed since the operation —nearly three years—is sufficient guarantee that the cure will be permanent, especially as the patient was addicted to the opium habit, and these patients are proverbially hard to cure of neuralgia, as the latter offers so good an excuse for resumption of their old habits.—Ex.

